# Pilot Study of [^11^C]HY-2-15: A Mixed Alpha-Synuclein and Tau PET Radiotracer

**DOI:** 10.3390/cells14151157

**Published:** 2025-07-26

**Authors:** Chia-Ju Hsieh, Dinahlee Saturnino Guarino, Anthony J. Young, Andrew D. Siderowf, Ilya Nasrallah, Alexander Schmitz, Carol Garcia, Ho Young Kim, Erin K. Schubert, Hsiaoju Lee, Joel S. Perlmutter, Robert H. Mach

**Affiliations:** 1 Department of Radiology, Perelman School of Medicine, University of Pennsylvania, Philadelphia, PA 19104, USA; chiahs@pennmedicine.upenn.edu (C.-J.H.); dinahlee.saturninoguarino@pennmedicine.upenn.edu (D.S.G.); anthony.young@pennmedicine.upenn.edu (A.J.Y.); ilya.nasrallah@pennmedicine.upenn.edu (I.N.); alexander.schmitz@pennmedicine.upenn.edu (A.S.); carol.garcia@pennmedicine.upenn.edu (C.G.); areas2001@kirams.re.kr (H.Y.K.); erinschu@pennmedicine.upenn.edu (E.K.S.); leehsi@pennmedicine.upenn.edu (H.L.); 2 Department of Neurology, Perelman School of Medicine, University of Pennsylvania, Philadelphia, PA 19104, USA; andrew.siderowf@pennmedicine.upenn.edu; 3 Department of Neurology, School of Medicine, Washington University, Saint Louis, MO 63110, USA; perlmutterjoel@wustl.edu

**Keywords:** α-synuclein, tau, progressive supranuclear palsy, multiple system atrophy, Parkinson’s disease, PET, radioligand

## Abstract

A novel brain positron emission tomography (PET) radioligand, [^11^C]HY-2-15, has potential for imaging alpha-synuclein aggregations in multiple system atrophy and misfolded tau proteins in tauopathies, based on its high binding affinity in disease brain tissue homogenates. Here, we demonstrate that [^3^H]HY-2-15 has the capability to bind to aggregated alpha-synuclein in multiple system atrophy brain and tau aggregations in progressive supranuclear palsy and corticobasal degeneration brain tissues via in vitro autoradiography study. A first-in-human pilot multicenter clinical study recruited a total of 10 subjects including healthy controls and patients with Parkinson’s disease, multiple system atrophy, or progressive supranuclear palsy. The study revealed that [^11^C]HY-2-15 has a relatively higher specific uptake in the pallidum and midbrain of patients with progressive supranuclear palsy. Total-body scans performed on the PennPET Explorer showed the radiotracer was cleared by renal excretion. However, the rapid metabolism and low brain uptake resulted in a limited signal of [^11^C]HY-2-15 in brain.

## 1. Introduction

Parkinsonism represents a constellation of symptoms manifested by different progressive neurodegenerative diseases but with different pathologies [1]. Two of these conditions include Parkinson’s disease (PD) and multiple system atrophy (MSA) that have abnormal alpha-synuclein (α-syn) aggregations in the brain. Aggregated α-syn accumulates in nerve cells of PD brain as Lewy neurites and Lewy bodies, whereas in MSA, aggregated α-syn accumulates in glial cells as glial cytoplasmic inclusions (GCIs). MSA includes two subtypes based on the predominant clinical symptoms—parkinsonian (MSA-P) and cerebellar (MSA-C) subtypes [2]. Two other parkinsonian conditions, progressive supranuclear palsy (PSP) and corticobasal degeneration (CBD), have abnormal 4-repeat (4R) [3,4] tau aggregations in different brain regions [5]. Therefore, a biomarker, such as a positron emission tomography (PET) radioligand, that can differentiate across diseases having similar symptoms, can potentially improve the accuracy of clinical diagnosis.

Several PET radioligands in clinical trials have been developed for imaging α-syn or tau. For imaging α-syn aggregation in patients with MSA, [^18^F]ACI-12589 has been reported as a promising radiotracer, which could be used to improve the clinical diagnosis to differentiate MSA from PD [6]. Another PET radiotracer for imaging α-syn aggregation that has been utilized in clinical trial is [^18^F]C05-05, which has shown promise in imaging α-syn aggregation in both PD and MSA patients [7]. In the development of tau PET radioligands, most radiotracers image tau aggregations in Alzheimer’s disease (AD) but have shown limited success in the non-AD tauopathies [8]. The tau radiotracer, [^18^F]APN-1607, has the potential to differentiate patients with PSP from those with MSA, PD, or AD [9,10].

Our group previously reported that [^11^C]HY-2-15 (compound [^11^C]4i in the publication) has favorable pharmacokinetic properties in PET imaging studies in healthy non-human primates [11]. In the in vitro binding assay study, using a tritium labeled compound, [^3^H]HY-2-15, revealed that HY-2-15 has high binding affinities for α-syn aggregations in MSA and PD postmortem brain tissues as well as for misfolded tau proteins in AD, PSP, and CBD brain tissue homogenates. Then, the [^3^H]HY-2-15 in vitro autoradiography studies focused on the synucleinopathies versus healthy control (HC) indicated that HY-2-15 has the potential to selectively detect GCIs in patients with MSA-P over Lewy bodies in patients with PD.

In this study, we extended the high-resolution autoradiography in vitro experiments by including both types of MSA and 4R tauopathy brain tissues. This demonstrated the capability of [^3^H]HY-2-15 to detect misfolded tau protein in PSP and CBD and the aggregated α-syn in both types of MSA in postmortem brain tissues. This was followed with a first-in-human pilot clinical study of [^11^C]HY-2-15 in HC and patients with PD, MSA, and PSP. The results of the first-in-human PET image study demonstrated a specific retention of [^11^C]HY-2-15 in the pallidum and midbrain of patients with PSP.

## 2. Materials and Methods

### 2.1. In Vitro Real-Time Autoradiography and Immunohistochemistry (IHC)

The [^3^H]HY-2-15 autoradiography studies were performed on postmortem brain sections from MSA-C, MSA-P, PD, dementia with Lewy bodies (DLB), CBD, PSP, and HC cases as follows. The postmortem tissue sections were acquired from the Center for Neurodegenerative Disease Research at the University of Pennsylvania, and the clinical demographic data is shown in Table S1. The formalin-fixed paraffin-embedded (FFPE) sections underwent deparaffination, rehydration, and epitope retrieval using a heat-induced epitope retrieval (HIER) step with citrate buffer (0.5 M, pH 6.0) for 1 h at 70 °C in a water bath. After 30 min of pre-incubation with PBS (pH 7.4), the binding reaction was initiated by incubating the sections with 10 nM [^3^H]HY-2-15 for 90 min at room temperature. Non-specific binding was determined on adjacent sections by adding 1 μM of unlabeled HY-2-15 to the 10 nM of [^3^H]HY-2-15. The reaction was terminated by washing the sections for 5 min in cold (4 °C) buffer three times and then dipping them once in cold distillated water. Slides were allowed to air-dry before being exposed and scanned in a real-time autoradiography instrument (BeaQuant, ai4R, Nantes, France) for 24 h. To assess tracer binding to α-syn and tau, we carried out staining for each of these markers in adjacent sections. Tissue sections were permeabilized with 0.1% Triton X-100 for 10 min, covered with hydrogen-peroxide blocking solution for 15 min, and immersed in blocking buffer (PBS + 10% goat serum + 1% BSA + 0.1% Tween 20) for 1 h at room temperature. Between each step, slides were washed with PBS Tween 20 (PBST) buffer three times per 5 min each. Afterwards, the sections were exposed overnight at 4 °C to the following primary antibodies: anti-Alpha-synuclein (phospho S129) antibody P-syn/81A and anti-Phospho-Tau (Ser202, Thr205) Monoclonal Antibody (AT8) (Table S2) at 1:500 dilution. Exposure to secondary antibody (Goat Anti-Mouse IgG H&L (HRP) (Table S3)) at 1:10,000 lasted 1 h at room temperature. Sections were washed again with PBST buffer, three times per 5 min each, visualized by developing them with DAB substrate for 10 min, and counterstained with Meyer’s hematoxylin dye. The sections were dehydrated, mounted in Limonene media, and cover slipped for microscopy. Images were captured with a Leica Aperio slide scanner (RRID:SCR_022420; Leica Biosystems; Deer Park, IL, USA) at 10× magnification. The PBS, Triton X-100, goat serum, BSA, and Tween 20 were purchased from Thermo Fisher Scientific (Waltham, MA, USA) or Sigma-Aldrich (Allentown, PA, USA).

### 2.2. Participants

The study was conducted in accordance with the Declaration of Helsinki and approved by the single Institutional Review Board at University of Pennsylvania (protocol code 853636 and date of approval 11 July 2023). Each university has its own eIND (University of Pennsylvania IND #167891; Washington University-Saint Louis IND #173078). The study was registered on ClinicalTrials.gov (NCT06032026). Informed consent was obtained from all participants in the study. A total of 10 participants were included in this study. Two HC, four PSP, one MSA-P, and three PD patients were recruited from the University of Pennsylvania and Washington University in Saint Louis.

### 2.3. Preparation of [^11^C]HY-2-15

According to the prior reported methods, [^11^C]HY-2-15 was radiosynthesized [11]. The radiochemical yield was 5.35 ± 2.42% and 312.83 ± 115.22 GBq/µmol of the specific activity at the end of the synthesis with the purity of 98.57 ± 1.60%.

### 2.4. Radiometabolism Study of [^11^C]HY-2-15

Radiometabolite analyses were performed on the 6 participants recruited from the University of Pennsylvania. Arterial blood samples were collected at 3, 8, 15, 30, and 60 min post [^11^C]HY-2-15 injection. Plasma samples were prepared for high-performance liquid chromatography (HPLC) by deproteination with acetonitrile at 1:1 by volume, centrifugation to separate supernatant, and filtering with a 0.45 μm syringe filter. HPLC analysis was performed on an Agilent 1260 Infinity Series HPLC (Agilent Technologies; Santa Clara, CA, USA) with an Agilent Zorbax Eclipse XDB-C18 column (150 mm × 4.6 mm, 5 μm pore size), a mobile phase of 0.1% trifluoroacetic acid buffer, and acetonitrile at a ratio of 73:27. HPLC elution fractions were collected and measured via a Wizard^2^ 2480 gamma counter (Perkin Elmer; Waltham, MA, USA).

### 2.5. PET Data Acquisition

All the participants underwent T1-weighted magnetic resonance (MR) scan to obtain anatomical information for facilitating spatial normalization and volumes of interest (VOIs) analyses.

#### 2.5.1. University of Pennsylvania

All the [^11^C]HY-2-15 PET/computed tomography (CT) scans performed at the University of Pennsylvania were conducted on the total-body PennPET Explorer [12]. A low-dose CT scan was performed for anatomical localization and attenuation correction followed by a 90 min or 120 min (12 × 10 s, 2 × 30 s, 4 × 60 s, 2 × 120 s, 3 × 180 s, 4 × 300 s and 8 × 600 s) dynamic PET image acquired in list-mode after a bolus [^11^C]HY-2-15 venous injection of 204.10 ± 92.18 MBq. A break was provided as needed during the dynamic scan. The PET image was reconstructed using time-of-flight list-mode ordered subsets expectation maximization (OSEM, 25 subsets) reconstruction algorithm. The reconstructed images had a matrix size of 300 × 300 × 712 and a voxel size of 2 × 2 × 2 mm^3^.

#### 2.5.2. Washington University in Saint Louis

The [^11^C]HY-2-15 PET/CT scans performed at Washington University in Saint Louis were conducted on a Siemens Vision 600 scanner. A low-dose CT scan was performed for anatomical localization and attenuation correction followed by a 120 min (36 × 5 s, 12 × 10 s, and 23 × 300 s) dynamic PET image acquired after [^11^C]HY-2-15 venous injection of 532.62 ± 180.77 MBq. The PET image was reconstructed using OSEM (8 iterations and 5 subsets) algorithm. The reconstructed images had a matrix size of 440 × 440 × 159 and a voxel size of 0.825 × 0.825 × 1.6456 mm^3^.

### 2.6. Image Analysis

The image analysis program, PMOD (version 4.2, PMOD Technologies Ltd., Zurich, Switzerland), was used to process and analyze all the images. Each dynamic brain PET image was motion-corrected by using the perfusion phase (1–6 min) image as the reference image. The perfusion phase PET image was co-registered to the corresponding T1-weighted MR image. The individual T1-weighted MR image was spatially normalized to the Montreal Neurologic Institute (MNI) MR-T1 brain template. Then, the spatial normalization parameters were applied to the corresponding dynamic brain PET image to form a spatially normalized brain PET image in the MNI template domain. Six VOIs were determined using the automated anatomical labeling (AAL) atlas including cerebral cortex (merged of frontal, temporal, parietal, and occipital), caudate, putamen, thalamus, pallidum, and cerebellar cortex. Additional three VOIs including midbrain, pons, and medulla were determined using the Amyloid Cortical Composite atlas in PMOD.

Standardized uptake value ratio (SUVR) was calculated for each VOI using cerebellar cortex as the reference region. Percent difference (%Difference) of SUVR to HC for each disease group was computed for all VOIs.

Additional VOIs of peripheral organs including heart wall, liver, gallbladder, spleen, spinal bone marrow, bilateral kidneys, and urinary bladder were manually delineated on the PET or CT image of the six scans obtained from the PennPET Explorer. Percent injected dose (%ID) was calculated for each peripheral organ for further biodistribution analysis.

## 3. Results

### 3.1. In Vitro Autoradiography

**Figure 1:**
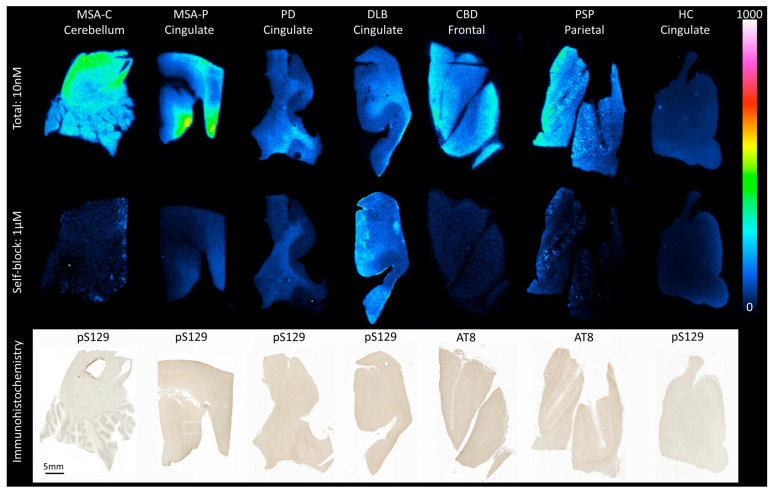
Autoradiography showing total binding using 10 nM [^3^H]HY-2-15 (**upper panel**) and non-specific binding (**middle panel**; determined with addition of 1 μM cold HY-2-15) of postmortem FFPE brain sections containing the cerebellum from MSA-C, the cingulate cortex from MSA-P, PD, DLB, and HC, the frontal cortex from CBD, and the parietal cortex from PSP cases. Autoradiography color/brightness threshold levels are expressed in counts (0–1000). Per MSA-C, MSA-P, PD, DLB, and HC cases, pS129 α-syn IHC (**lower panel**) was performed on adjacent sections as pathology reference, while for CBD and PSP, AT8 IHC was carried out. pS129, anti-phospho129 α-syn antibody; AT8, anti-phosphorylated tau antibody. Scale bars: 5 mm. MSA-C, multiple system atrophy—cerebellar subtype; MSA-P, multiple system atrophy—parkinsonian subtype; PD, Parkinson’s disease; DLB, dementia with Lewy bodies; CBD, corticobasal degeneration; PSP, progressive supranuclear palsy; and HC, healthy control.

Target engagement of [^3^H]HY-2-15 was evaluated using in vitro autoradiography in brain sections from MSA-C, MSA-P, PD, DLB, CBD, PSP, and HC in conjunction with α-syn PS129 and tau AT8 IHC (Figure 1 and Figure S1). Visual assessment of the total binding for [^3^H]HY-2-15 revealed a clear autoradiographic signal in the cerebellar white matter of an MSA-C case and in the cingulate cortex of an MSA-P case. The specific binding of [^3^H]HY-2-15 in MSA-P and MSA-C sections overlapped with α-syn pS129 neuropathology (Figure 1 and Figure S1A–C). Both MSA-P and MSA-C tissue sections had a pure high level of α-syn pathology (Table S1). A moderate binding level of [^3^H]HY-2-15 was shown in the CBD frontal and PSP parietal section (Figure 1). Both CBD and PSP brain tissue contain a high level of tau but are negative of α-syn pathology (Table S1). Co-localization of the [^3^H]HY-2-15 autoradiographic signal with tau AT8 IHC was observed in PSP in regions where tufted astrocytes were present (Figure S1G–I). Whereas a low level of [^3^H]HY-2-15 binding was observed in the cingulate cortex in PD and DLB (Figure 1 and Figure S1D–F). Both PD and DLB tissue sections had high levels of α-syn and Aβ pathologies (Table S1). There was no binding of [^3^H]HY-2-15 in the cingulate gyrus section of HC with negative pathology of tau, α-syn, and Aβ (Figure 1 and Figure S1J–L and Table S1).

### 3.2. Human Brain Images of [^11^C]HY-2-15

**Figure 2:**
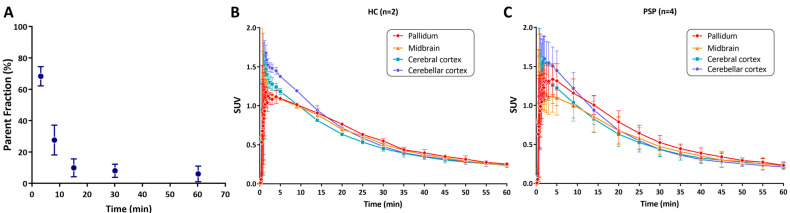
(**A**) Parent fraction of [^11^C]HY-2-15 from arterial blood samples. Time–activity curves of pallidum, midbrain, cerebral cortex, and cerebellar cortex in SUV for (**B**) HC and (**C**) PSP. Data is presented as mean ± standard deviation. HC, healthy control; PSP, progressive supranuclear palsy; and SUV, standardized uptake value.

The demographic data of all the participants is summarized in Table 1. A subset of participants including one HC, one PD, one MSA-P, and three PSP had arterial blood sampling for metabolite analysis. The free fraction of [^11^C]HY-2-15 was 0.5 ± 0.2%, and [^11^C]HY-2-15 was rapidly metabolized, with parent fractions of 68.33 ± 6.18%, 9.90 ± 5.69%, and 8.30 ± 4.24% at 3, 15, and 30 min post radioligand injection, respectively (Figure 2A). The radio metabolites consisted of one major polar metabolite (Figure S2).

The uptake of [^11^C]HY-2-15 peaked rapidly at approximately 2 min post tracer injection, followed by a rapid washout with an average clearance half-time of 18.85 ± 4.03 min (Figure 2 and Figure S3). The SUV converted to lower than 0.5 at around 30 min post [^11^C]HY-2-15 injection in all the brain regions.

**Figure 3:**
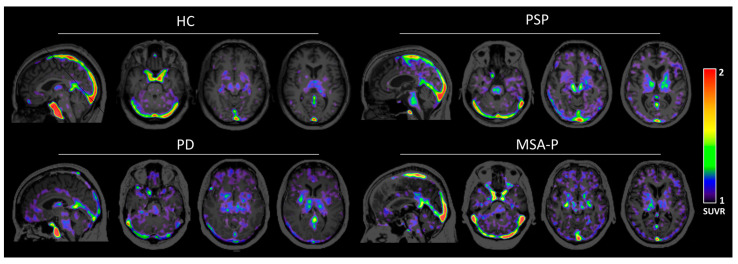
Representative 15–35 min [^11^C]HY-2-15 SUVR image with a whole brain mask of HC, PSP, PD, and MSA-P in the sagittal and axial views of cerebellum, midbrain, and basal ganglia. HC, healthy control; PSP, progressive supranuclear palsy; PD, Parkinson’s disease; MSA-P, multiple system atrophy—parkinsonian subtype; and SUVR, standardized uptake value ratio.

**Figure 4:**
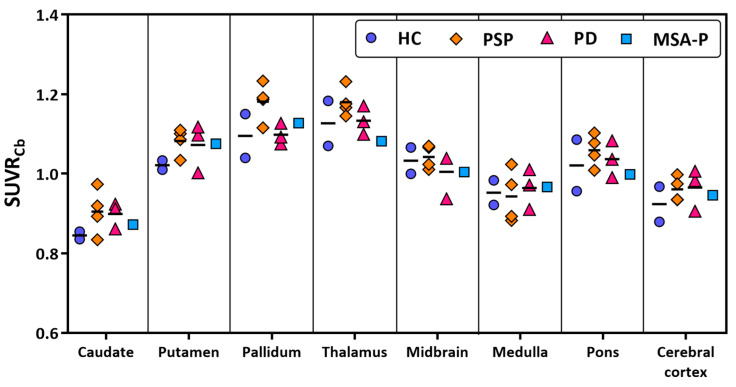
Scatter plot of brain regional [^11^C]HY-2-15 SUVRs in HC, PSP, PD, and MSA-P. HC, healthy control; PSP, progressive supranuclear palsy; PD, Parkinson’s disease; MSA-P, multiple system atrophy—parkinsonian subtype; and SUVR_Cb_, standardized uptake value ratio using cerebellar cortex as reference region.

The brain uptake washed out to background levels at approximately 40 min post tracer injections (Figure 2B,C and Figure S3), likely due to marked reduction in the parent fraction of [^11^C]HY-2-15 to less than 10% after 30 min post tracer injection (Figure 2A). Hence, the image time window, 15 to 35 min, was selected to calculate regional SUVR for further HC and disease group comparison. Cerebellar cortex, the region with minimal pathologic involvement in PD, MSA-P, or PSP, was selected as the reference region to calculate SUVRs. The representative SUVR image for each group on the scale of one to two to show the specific binding of [^11^C]HY-2-15 is displayed in Figure 3. The individual full scale SUVR image for all 10 participants is shown in Figure S4. HC and MSA-P had low uptakes of [^11^C]HY-2-15 in cortical and subcortical regions (Figure 3, Figure 4, and Figure S4). In two of the three PD patients, a slightly higher specific binding of [^11^C]HY-2-15 in putamen (4.94 ± 6.00 %Difference; Table S4, Figure 3, Figure 4, and Figure S4) was observed. A relative higher [^11^C]HY-2-15 retention was observed in three of the PSP patients (Figure 3, Figure 4, and Figure S4) at midbrain, pallidum, thalamus, and pons. Only putamen (5.93% ± 3.31%) and pallidum (7.90% ± 4.45%) showed %Difference higher than 5% as compared to HC (Table S4). None of the regions showed statistically significant differences between HC and disease groups in the nonparametric Mann–Whitney U or Kruskal–Wallis test. A high radio signal was observed in the middle and posterior venous sinuses in all the subjects.

### 3.3. Biodistribution of [^11^C]HY-2-15

**Figure 5:**
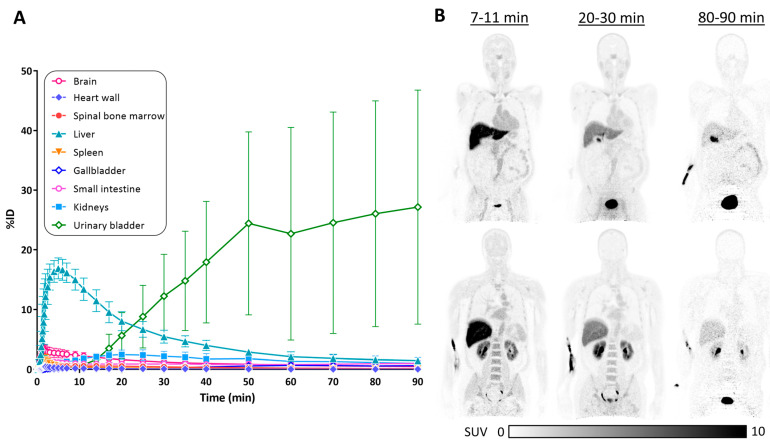
Biodistribution of [^11^C]HY-2-15. (**A**) Time–activity curves of %ID in brain and peripheral organs. (**B**) Total-body SUV image of 7–11 min, 20–30 min, and 80–90 min post [^11^C]HY-2-15 injection in the two different coronal views of a healthy control subject. SUV, standardized uptake value; and %ID, percent injected dose.

An illustration of the total-body biodistribution PET image of an HC subject and the time–activity curve of %ID are shown in Figure 5. A high initial uptake of liver (9.43 ± 2.08 %ID) was observed in the first 20 min post [^11^C]HY-2-15 injection and cleared out to lower than 5 %ID at approximately 40 min (Table S5). The radio activities started to accumulate in the urinary bladder at 20 min (3.59 ± 2.31 %ID) and increased to 26.85 ± 19.32 %ID at the end of the scan (Table S5). The %ID of brain at the peak uptake of [^11^C]HY-2-15 was 3.15 ± 0.88%, then decreased to lower than 2% at 14 min post tracer injection. The radio activities were maintained at a low level with %ID lower than 1% over the 90 min dynamic scan in the heart wall, spinal bone marrow, spleen, gallbladder, and small intestine (Table S5).

## 4. Discussion

The radioligand, [^11^C]HY-2-15, has been identified as a potential PET radiotracer for imaging α-syn in MSA and 4R tau in the 4R tauopathies [11]. In this study, we extended the in vitro autoradiography studies in both tauopathies and synucleinopathies to demonstrate the selective binding of [^3^H]HY-2-15 in human brain tissues, followed by a small pilot study in human subjects with [^11^C]HY-2-15 PET imaging. In the autoradiography study, the high specific binding in both types of MSA, but low binding in PD or DLB brain tissues, and no binding in HC were consistent with our previous results. The high specific binding in CBD and PSP brain tissues was also consistent with the previous study of the [^3^H]HY-2-15 in vitro tissue homogenate binding assay showing a high potency of binding in PSP and CBD. These results indicated that HY-2-15 has the potential to serve as a PET radioligand to image both MSA and tauopathies patients.

In the PET imaging studies of [^11^C]HY-2-15 in human subjects, brain regions including putamen, pallidum, thalamus, midbrain, and pons showed a moderate level of [^11^C]HY-2-15 retention for patients with PSP. This is consistent with the pathological spreading pattern of 4R tau in PSP [4,13] and the PET image uptake pattern in patients with PSP imaging with 3R/4R tau mixed radiotracers [9,14]. Although there was no statistically significant difference between PSP and HC in the regional SUVR comparison, the increase in [^11^C]HY-2-15 uptake in the tau pathology regions and low uptake in cerebral cortex indicate that [^11^C]HY-2-15 has potential to image aggregated 4R tau in patients with PSP. The PET imaging results were also consistent with what was observed with [^3^H]HY-2-15 in radioligand binding assays in tissue homogenates and autoradiography studies with sections of PSP brain. The low statistical power in the PET studies was due to the low sample size in the current study.

The observation of low brain [^11^C]HY-2-15 retention for PD patients is consistent with what was observed with [^3^H]HY-2-15 autoradiography studies. Both [^3^H]HY-2-15 autoradiography in the current study and nuclear emulsion autoradiography from the previous report [11] showed low specific binding in autopsy-confirmed postmortem PD brain section with Lewy body pathology.

In the PET study of the patient with MSA-P, a minor uptake of [^11^C]HY-2-15 was observed in the midbrain and choroid plexus. There was no observation of specific uptake in the putamen and cerebellar peduncle—the critical brain regions with high α-syn pathologies in MSA-P [2,15], and regions having a high retention with the α-syn PET radioligands, [^18^F]C05-05 [7] and [^18^F]ACI-12589 [6]. This represents a discrepancy in the binding of HY-2-15 to MSA brain between in vitro and in vivo studies. That is, [^3^H]HY-2-15 bound to MSA tissue in both radioligand tissue homogenate binding assays and autoradiography studies in brain sections, whereas [^11^C]HY-2-15 did not show an uptake in regions having high α-syn burden in the PET imaging studies. The reason for the discrepancy is not known. Although the binding affinities of [^3^H]HY-2-15 in MSA and PSP brain tissue homogenates are similar [11], given the low density of α-syn in MSA brain, the binding affinity might not be potent enough for [^11^C]HY-2-15 to image α-syn aggregates in vivo. Additional studies in patients with MSA will be needed to confirm this. Of course, clinical diagnosis of MSA-P is not perfect, so potential misdiagnosis could contribute to this lack of specificity in this small study.

The initial brain uptake of [^11^C]HY-2-15 at peak was approximately 3 %ID with an SUV of approximately 1.5. As compared to the range of 5 to 10 %ID in the brain for the currently used radioligands for imaging Aβ and tau aggregations [16,17,18,19], the peak brain uptake of [^11^C]HY-2-15 might be lower than what is required for imaging α-syn and tau aggregates in vivo. The low brain uptake and rapid metabolism of [^11^C]HY-2-15 resulted in the observation of high uptake in venous sinuses and limited specific binding in the brain.

The radiotracer rapidly accumulated in the urinary bladder at 15 min post [^11^C]HY-2-15 injection, the parent fraction decreased to 10% with one major polar radiolabeled metabolite, and rapidly washed out from the liver. These results suggest that the radiometabolite of [^11^C]HY-2-15 is very polar, likely from the hydrolysis of the amid bond thereby promoting renal excretion. Although there was high initial uptake of [^11^C]HY-2-15 in the liver, high liver uptake is often seen with PET radiotracers since this represents a major site of tracer metabolism. The relatively low dose administered in our studies (200–300 MBq) and the short half-life of carbon-11 (20.4 min) are not expected to cause any radiation damage to the liver.

## 5. Conclusions

[^3^H]HY-2-15 has the capability to bind to α-syn and tau aggregations in MSA, PSP and CBD brain in vitro. The pilot human PET imaging studies suggest that [^11^C]HY-2-15 has the potential to image 4R tauopathy in patients with PSP. However, the low brain uptake and rapid metabolism resulted in the low signal-to-noise ratio in the brain. Studies to develop second generation radioligands with more desirable properties for imaging α-syn or tau aggregations are currently ongoing.

## Figures and Tables

**Table 1:** Demographics of the participants in [^11^C]HY-2-15 studies.

	HC	PSP	PD	MSA-P
Sample size	2	4	3	1
Sex (Female/Male)	1/1	1/3	1/2	0/1
Age (Year)	71.5 ± 3.5	62.8 ± 5.0	57.0 ± 6.9	72
Hoehn–Yahr Stage	-	3.5 ± 0.6	2.7 ± 1.2	2
UPDRS III	4.0 ± 5.7	42.5 ± 24.4	58.7 ± 22.1	43

Data is presented as mean ± standard deviation. HC, healthy control; PSP, progressive supranuclear palsy; PD, Parkinson’s disease; MSA-P, multiple system atrophy—parkinsonian subtype; and UPDRS, Unified Parkinson Disease Rating Scale.

## Data Availability

The original contributions presented in this study are included in the article and Supplementary Materials. Further inquiries can be directed to the corresponding author.

## Supplementary Materials

The following supporting information can be downloaded at https://www.mdpi.com/article/10.3390/cells14151157/s1: Table S1: Selected demographics of cases used for autoradiography and immunohistochemistry; Table S2: Primary antibody selected for immunohistochemistry; Table S3: Secondary antibody selected for immunohistochemistry; Table S4: Brain regional 15–35 min SUVRs of each group and %Difference to HC; Table S5: The %ID of brain and peripheral organs in every 10 min time duration of 90 min dynamic scan. Figure S1: The [^3^H]HY-2-15 autoradiography (ARG) in the (**A**) cingulate cortex of MSA-P, (**D**) cingulate cortex of DLB, (**G**) parietal cortex of PSP, and (**J**) cingulate cortex of HC cases. IHC of α-syn pS129 co-localization of (**B**,**C**) MSA-P, (**E**,**F**) DLB, and (**K**,**L**) HC tissues. IHC of AT8 co-localization of (**H**,**I**) PSP tissue. Magnifications of pS129 or AT8 IHC are shown from areas indicated by squares. Scale bars: 5 mm (**B**,**E**,**H**,**K**) and (**C**,**F**,**I**,**L**) 100 μm; Figure S2: HPLC radiochromatograms of arterial plasma sampled at 3, 8, and 15min post [^11^C]HY-2-15 injection measured via an in-line radiodetector (Posi-RAM; LabLogic Systems, Chantilly, VA). Parent compound is shown in blue, radiometabolites are shown in red and green.; Figure S3: Full 120 min time–activity curves of caudate, putamen, pallidum, thalamus, midbrain, pons, medulla, cerebral cortex, and cerebellar cortex in SUV for (**A**) HC, (**B**) PSP, (**C**) PD, and (**D**) MSA-P. Data was presented as mean ± standard deviation; Figure S4: Individual 15–35 min SUVR image of all the HC, PSP, PD, and MSA-P participants in the sagittal and axial views of cerebellum, midbrain, and basal ganglia.

## Abbreviations

The following abbreviations are used in this manuscript:

PD	Parkinson’s disease
MSA	Multiple system atrophy
α-syn	Alpha-synuclein
GCI	Glial cytoplasmic inclusion
PSP	Progressive supranuclear palsy
CBD	Corticobasal degeneration
4R	4-repeat
PET	Positron emission tomography
AD	Alzheimer’s disease
HC	Healthy control
IHC	Immunohistochemistry
DLB	Dementia with Lewy bodies
HPLC	High-performance liquid chromatography
MR	Magnetic resonance
VOI	Volume of interest
CT	Computed tomography
MNI	Montreal Neurologic Institute
AAL	Automated anatomical labeling
SUVR	Standardized uptake value ratio
%ID	Percent injected dose
