# Supplementary material for: Pilot Study of [11C]HY-2-15: A Mixed Alpha-Synuclein and Tau PET Radiotracer
Source: Cells. 2025 Jul 26;14(15):1157. doi: 10.3390/cells14151157 (PMC12346455; doi:10.3390/cells14151157)
Supplement: Supplementary file 1 [file cells-14-01157-s001.zip › cells-3705333-supplementary.pdf]

cells

MDPI

Article

Pilot Study of [^{11}C]HY-2-15: A Mixed Alpha-Synuclein and Tau PET Radiotracer

Chia-Ju Hsieh [1], Dinahlee Saturnino Guarino [1], Anthony J. Young [1], Andrew D. Siderowf [2], Ilya Nasrallah [1], Alexander Schmitz [1], Carol Garcia [1], Ho Young Kim [1], Erin K. Schubert [1], Hsiaoju Lee [1], Joel S. Perlmutter [3] and Robert H. Mach [1,*]

[1] Department of Radiology, Perelman School of Medicine, University of Pennsylvania, Philadelphia, PA 19104, USA; chiahs@pennmedicine.upenn.edu (C.-J.H.); dinahlee.saturninoguarino@pennmedicine.upenn.edu (D.S.G.); anthony.young@pennmedicine.upenn.edu (A.J.Y.); ilya.nasrallah@pennmedicine.upenn.edu (I.N.); alexander.schmitz@pennmedicine.upenn.edu (A.S.); carol.garcia@pennmedicine.upenn.edu (C.G.); areas2001@kirams.re.kr (H.Y.K.); erinschu@pennmedicine.upenn.edu (E.K.S.); leehsi@pennmedicine.upenn.edu (H.L.)
[2] Department of Neurology, Perelman School of Medicine, University of Pennsylvania, Philadelphia, PA 19104, USA; andrew.siderowf@pennmedicine.upenn.edu
[3] Department of Neurology, School of Medicine, Washington University, Saint Louis, MO 63110, USA; perlmutterjoel@wustl.edu
* Correspondence: rmach@pennmedicine.upenn.edu

Table S1. Selected demographics of cases used for autoradiography and immunohistochemistry.................................. 2

Table S2. Primary antibody selected for immunohistochemistry. 2

Table S3. Secondary antibody selected for immunohistochemistry. 2

Figure S1. [^{3}H]HY-2-15 autoradiography (ARG) in the (A) cingulate cortex of MSA-P, (D) cingulate cortex of DLB, (G) parietal cortex of PSP, and (J) cingulate cortex of HC cases. IHC of α-syn pS129 co-localization of (B and C) MSA-P, (E and F) DLB, and (K and L) HC tissues.. 3

Figure S2. HPLC radiochromatograms of arterial plasma sampled at 3-, 8- and 15-min post [^{11}C]HY-2-15 injection. Parent compound is shown in blue, radiometabolites are shown in red and green. 4

Figure S3. Full 120-min time-activity curves of caudate, putamen, pallidum, thalamus, midbrain, pons, medulla, cerebral cortex, and cerebellar cortex in SUV for (A) HC, (B) PSP, (C) PD, and (D) MSA-P... 4

Figure S4. Individual 15-35 min SUVR image of all the HC, PSP, PD, and MSA-P participants in the sagittal and axial views of cerebellum, midbrain and basal ganglia. 5

Table S4. Brain regional 15-35 min SUVRs of each group and %Difference to HC 5

Table S5. %ID of brain and peripheral organs in every 10-min time duration of 90-min dynamic scan........................... 6

Table S1. Selected demographics of cases used for autoradiography and immunohistochemistry.

Diagnosis	Gender (M/F)	Age of Onset (Years)	Age at Death (Years)	Tau	Aβ	α-syn	TDP-43	Region used
PSP	F	75	80	3+	2+	0	0	Parietal
CBD	M	80	86	3+	0	0	0	Frontal
MSA-C	M	60	68	0	0	3+	0	Cerebellum
MSA-P	M	51	59	0	0	3+	0	Cingulate cortex
PD	M	67	83	3+	3+	3+	0	Cingulate cortex
DLB	M	70	73	1+	3+	3+	0	Cingulate cortex
HC	M	-	66	0	0	0	0	Cingulate gyrus

Table S2. Primary antibody selected for immunohistochemistry.

Primary Antibodies	Source/Cat No	Host and clonality	Antibody dilution used	Incubation time
Phospho-Tau (Ser202, Thr205) Monoclonal Antibody (AT8)	Thermo Fisher Scientific #MN1020	Mouse monoclonal	1:500	Overnight at 4 ºC
Anti-Alpha-synuclein (phospho S129) antibody P-syn/81A	Abcam/ab184674)	Mouse monoclonal	1:500	Overnight at 4 ºC

Table S3. Secondary antibody selected for immunohistochemistry.

Secondary Antibody	Source/Cat No	Host and clonality	Antibody dilution used	Incubation time
Goat Anti-Mouse IgG H&L	Abcam/ab205719	Goat polyclonal	1:10000	1 h at ambient temperature

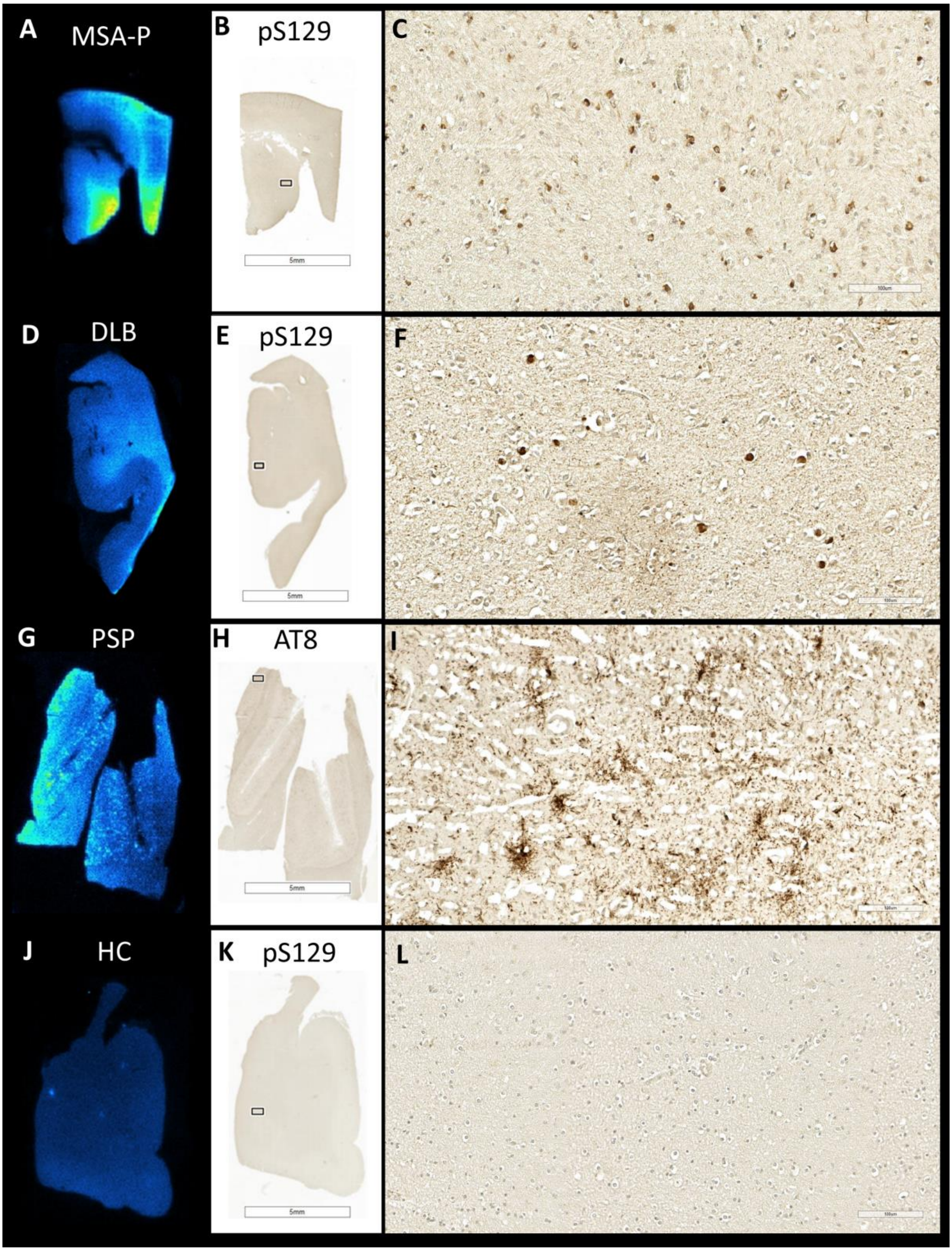

Figure S1. The [^{3}H]HY-2-15 autoradiography (ARG) in the (**A**) cingulate cortex of MSA-P, (**D**) cingulate cortex of DLB, (**G**) parietal cortex of PSP, and (**J**) cingulate cortex of HC cases. IHC of α-syn pS129 co-localization of (**B**,**C**) MSA-P, (**E**,**F**) DLB, and (**K**,**L**) HC tissues. IHC of AT8 co-localization of (**H**,**I**) PSP tissue. Magnifications of pS129 or AT8 IHC are shown from areas indicated by squares. Scale bars: 5 mm (**B**,**E**,**H**,**K**) and (**C**,**F**,**I**,**L**) 100 μm

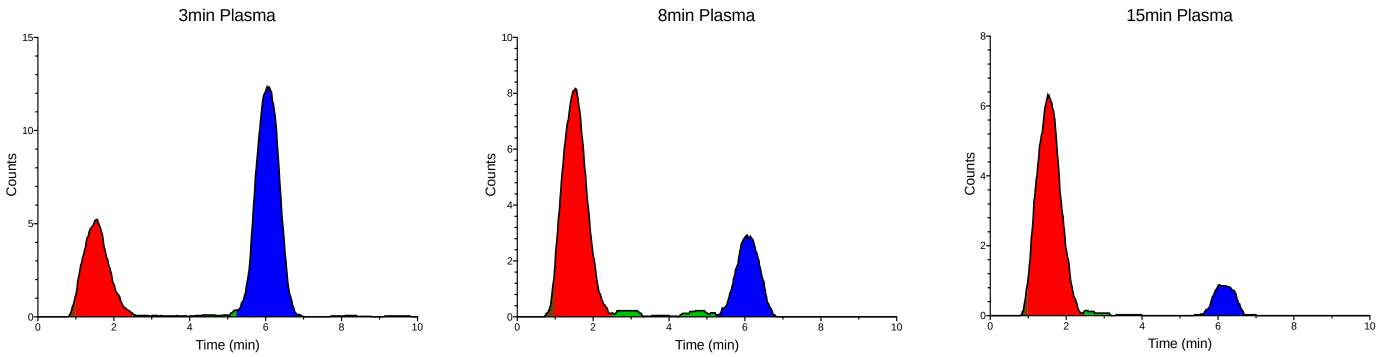

Figure S2. HPLC radiochromatograms of arterial plasma sampled at 3, 8 and 15 min post [^{11}C]HY-2-15 injection, measured via an in-line radiodetector (Posi-RAM; LabLogic Systems, Chantilly, VA). Parent compound is shown in blue, radiometabolites are shown in red and green.

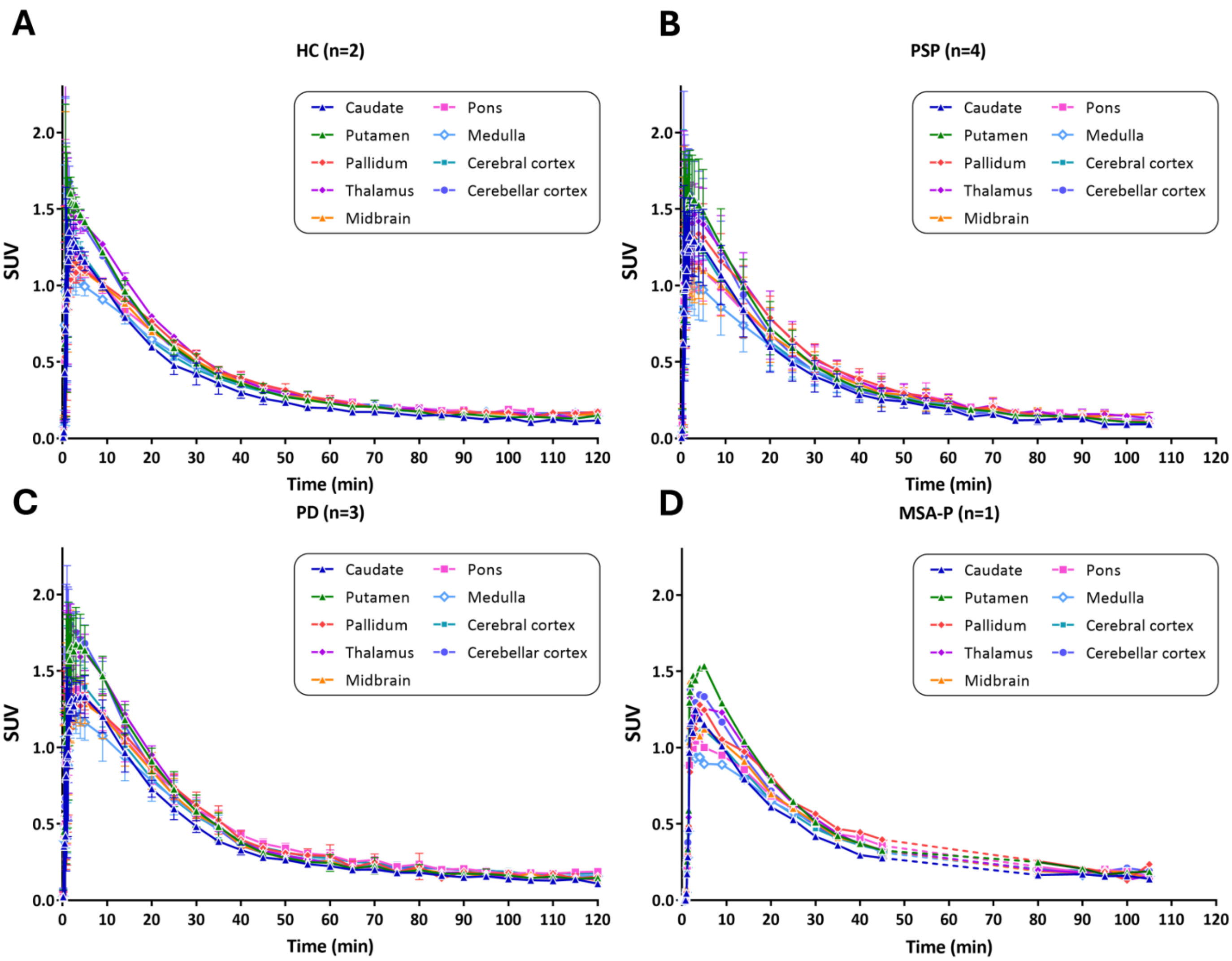

Figure S3. Full 120 min time-activity curves of caudate, putamen, pallidum, thalamus, midbrain, pons, medulla, cerebral cortex, and cerebellar cortex in SUV for (A) HC, (B) PSP, (C) PD, and (D) MSA-P. Data was presented as mean ± standard deviation.

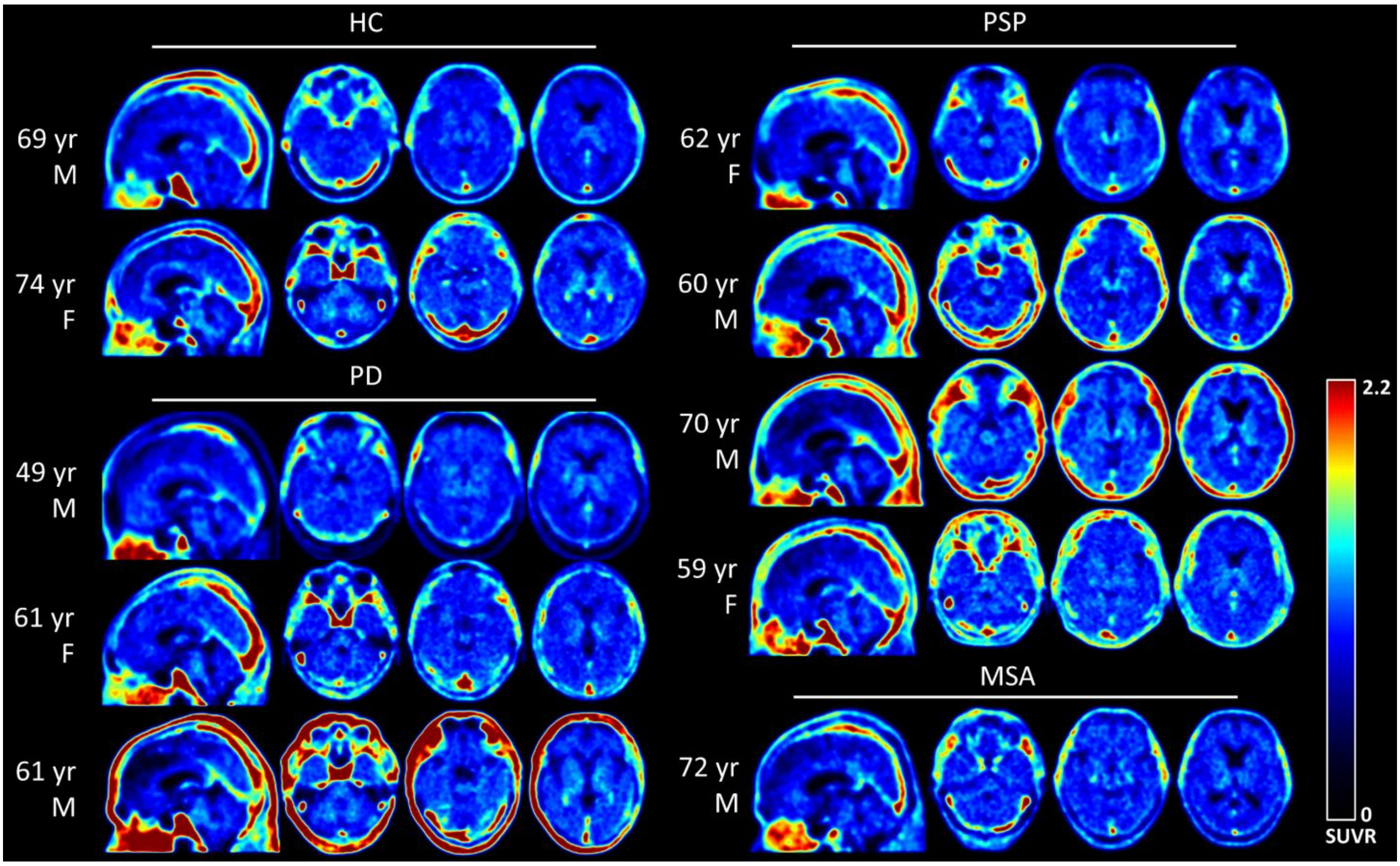

Figure S4. Individual 15-35 min SUVR image of all the HC, PSP, PD, and MSA-P participants in the sagittal and axial views of cerebellum, midbrain, and basal ganglia.

Table S4. Brain regional 15-35 min SUVRs of each group and %Difference to HC

	HC	PSP		PD		MSA-P	
Regions	SUVR	SUVR	%Difference	SUVR	%Difference	SUVR	%Difference
Caudate	0.85 ± 0.01	0.91 ± 0.06	7.10 ± 6.85	0.89 ± 0.04	6.40 ± 3.92	0.87	3.23
Putamen	1.02 ± 0.02	1.08 ± 0.03	5.93 ± 3.31	1.06 ± 0.08	4.94 ± 6.00	1.08	5.27
Pallidum	1.09 ± 0.08	1.18 ± 0.05	7.90 ± 4.45	1.08 ± 0.01	0.28 ± 2.42	1.13	2.97
Thalamus	1.13 ± 0.08	1.18 ± 0.04	4.71 ± 3.27	1.11 ± 0.02	0.61 ± 3.18	1.08	-3.98
Midbrain	1.03 ± 0.05	1.04 ± 0.03	0.92 ± 2.87	0.99 ± 0.07	-2.74 ± 5.66	1.00	-2.78
Medulla	0.95 ± 0.04	0.94 ± 0.07	-1.01 ± 7.04	0.96 ± 0.07	1.22 ± 5.31	0.97	1.49
Pons	1.02 ± 0.09	1.06 ± 0.04	3.71 ± 3.97	1.01 ± 0.03	1.53 ± 4.52	1.00	-2.23
Cerebral cortex	0.92 ± 0.06	0.96 ± 0.03	4.00 ± 3.38	0.96 ± 0.07	4.48 ± 5.69	0.95	2.40

Table S5. The %ID of brain and peripheral organs in every 10 min time duration of 90 min dynamic scan

Time (min)	Brain	Heart wall	Spinal bone marrow	Liver	Spleen	Gallbladder	Small intestine	Kidneys	Urinary bladder
0-11	2.09 ± 0.57	0.25 ± 0.13	0.31 ± 0.08	9.18 ± 2.54	0.73 ± 0.30	0.08 ± 0.07	0.97 ± 0.42	0.99 ± 0.24	0.18 ± 0.10
11-20	1.81 ± 0.42	0.13 ± 0.08	0.55 ± 0.25	9.68 ± 1.71	0.29 ± 0.14	0.09 ± 0.08	0.90 ± 0.28	2.30 ± 0.65	3.59 ± 2.31
20-30	1.32 ± 0.26	0.09 ± 0.06	0.45 ± 0.22	6.07 ± 1.19	0.21 ± 0.10	0.16 ± 0.13	0.90 ± 0.32	2.28 ± 0.84	10.52 ± 6.12
30-40	1.03 ± 0.16	0.07 ± 0.05	0.36 ± 0.18	4.47 ± 0.99	0.16 ± 0.08	0.29 ± 0.21	0.88 ± 0.39	1.94 ± 0.85	16.07 ± 8.75
40-50	0.89 ± 0.12	0.06 ± 0.04	0.33 ± 0.16	3.18 ± 0.72	0.13 ± 0.05	0.45 ± 0.17	0.73 ± 0.50	1.62 ± 1.06	21.79 ± 13.11
50-60	0.71 ± 0.13	0.03 ± 0.02	0.19 ± 0.12	2.55 ± 1.17	0.09 ± 0.05	0.59 ± 0.13	0.88 ± 0.42	1.34 ± 0.95	21.23 ± 15.62
60-70	0.63 ± 0.11	0.02 ± 0.01	0.15 ± 0.10	2.18 ± 0.96	0.08 ± 0.05	0.57 ± 0.21	0.95 ± 0.46	1.21 ± 0.99	23.44 ± 16.26
70-80	0.56 ± 0.10	0.02 ± 0.01	0.12 ± 0.07	1.91 ± 0.84	0.08 ± 0.04	0.54 ± 0.19	1.02 ± 0.48	1.06 ± 0.98	23.81 ± 16.73
80-90	0.49 ± 0.09	0.02 ± 0.01	0.11 ± 0.06	1.48 ± 0.60	0.07 ± 0.04	0.63 ± 0.14	0.98 ± 0.54	1.05 ± 1.03	26.85 ± 19.32
